# Female Genital Mutilation, Cutting, or Circumcision

**DOI:** 10.1155/2013/240421

**Published:** 2013-11-27

**Authors:** Johanne Sundby, Birgitta Essén, R. Elise B. Johansen

**Affiliations:** ^1^Institute of Health and Society, University of Oslo, Norway; ^2^Department of Women's and Children's Health, International Maternal and Child Health (IMCH), Uppsala University, Sweden; ^3^Norwegian Centre for Violence and Traumatic Stress Studies (NKVTS), Norway

Female genital mutilation (FGM), female genital cutting, or female circumcision of women, the theme addressed in this special issue has many terms. The short form acronym FGM is understood by most, and it does contain the notion that we are talking about a traditional practice that is harmful. The practice affects women in diaspora as well as African countries, and men are involved as decision makers and attitude changers. Cutting is a neutral term, and circumcision is a more traditional terminology. Each term carries a certain value. But the practice is the same regardless of name.

In order to understand the tradition, assist women who have undergone it, and promote action against it, it is important to have solid knowledge. This knowledge is partly medical and partly social. Thus, research based on a multitude of methods is warranted. This special issue is indeed a combination of social science and medical research on different aspects of the practice, that is also a genital health hazard for women.

The special issue addresses FGM from different angles and geographical places. Somalis are heavily affected as well with the most extensive form of FGM, and several studies presented are from Somalia or about Somalis elsewhere. There is a paper on health provider issues from Somalia that reveals how health workers may view the practice in a country where cutting is the norm (Lazar et al.). The other side of the issue in Somaliland, the care needed by clients, is described in another paper (Fried et al.). But also in West Africans, where most practice less extensive forms of FGM, its gender aspects is addressed, as the studies from the Gambia and Sierra Leone demonstrate (Kaplan et al. and Bjälkander et al.). In Sierra Leone, as in other countries, there are some difficulties encountered in how to assess the types and magnitude of FGM.

The phenomenon of FGM is important to gynaecologists for several reasons. Firstly, as it is a harmful practice that affects women, OB/GYN professionals may want to participate in initiatives that lead to its abandonment. As this is a topic that many people feel strongly about, some work on but fewer actually know how to handle, several articles in this issue may assist those who want to do their share in defining appropriate community interventions that they may want to engage in. The review of strategies presented here and the paper on attitude change are important contributions to the know-how literature. Some may think that harm reduction is a way forward, and others claim that full abandonment is the only way (Gele et al.).

Secondly, as this harmful practice is associated with health risks that OB/GYN professionals have to manage, many OB/GYN face problems when delivering babies of women who have undergone FGM, yet others have to provide different types of care to alleviate the problem, especially the obstetric consequences and their management. This special issue provides evidence for the harmful effects (Berg, Underland). As there may be an increased attempt to medicalize the cutting, and requests for doctors to involve themselves in “harm reduction” strategies, it is important that OB/GYN and nursing professionals are also fully aware of the reasons for wanting to be cut, male and female resistance to change, and knowledge about how harmful this practice is (Isman et al.). 

The issue of harm reduction is indeed controversial, as the bottom line message is to end the practice altogether. If one thinks that taking one step at a time and involving professionals in the procedure will eventually eliminate the practice, it may violate the very reason for medical people to practice “do no harm.” This is an issue that should be transparent and heavily debated everywhere. To perform FGM for “harm reduction” may as well turn into yet another profit operation for some health workers, because consumers ask for it. To have solid evidence on the damage done to women is therefore important. The systematic meta-analysis paper in this issue is the best of that knowledge (Johansen et al.). 

This special issue tries to link some evidence and experience from the social sciences into clinical obstetrics and gynaecology. We, as OB/GYN doctors, know that culture, social position, and gender also form our work environments, cause disease and complications are determinants for choices we and our patients make. Therefore, we hope you will enjoy this issue and join the growing group of colleagues who take this practice seriously as a step to a better health for girls and women everywhere.

*Johanne Sundby* *Johanne Sundby* *R. Elise B. Johansen* *R. Elise B. Johansen* *Birgitta Essén* *Birgitta Essén*

